# Early Childhood Caries in Peru

**DOI:** 10.3389/fpubh.2019.00337

**Published:** 2019-11-15

**Authors:** Jorge L. Castillo, Camila Palma, Ailín Cabrera-Matta

**Affiliations:** Department of Dentistry for Children and Adolescents, Universidad Peruana Cayetano Heredia, Lima, Peru

**Keywords:** early childhood caries, dental caries, Peru, oral health education, oral health children, oral health related quality of life

## Abstract

Early Childhood Caries (ECC) is a global oral health problem, and Peru may be one of the countries with high prevalence of untreated ECC in South America. In this study, we constructed an epidemiologic profile of ECC in Peru through a comprehensive review of published data. The prevalence of ECC, risk factors for it, its impact on child development, and public oral health interventions on ECC have been included. The study revealed extremely high rates of ECC in Peru and significant oral-health disparities. Risk factors for ECC were poverty, high sugar consumption, and low oral health literacy. However, the number of studies is limited and their quality questionable. Oral health has not received high public-health priority in Peru. However, in recent years, new regulations and evidence-based documents (the first Clinical Practice Guideline for the Prevention, Diagnosis, and Management of Caries in Children; the Guideline for Children's healthy Growth and Development; the Law on Healthy Diet; and the Manual on Food Advertising) give hope for the future of infants' oral health in the nation.

## Overview of Peru

Peru is the third largest country in South America, located in the western side of the continent, facing the Pacific Ocean. Peru is divided into three regions: the Andean region; the Amazon jungle; and the coast, facing the Pacific Ocean. The three regions have unique geography, culture, ethnicity, and economic development.

The estimated population of Peru was 31,237,385 inhabitants according to the most recent census (1). Children under 14 years of age constituted 26.4% of the population, and the total population of children 0–5 years old was 3,405,500. Peru is considered a developing country, with upper-middle income economy, according to the World Bank (2).

This article presents an overview of the status of early childhood caries (ECC) in Peru, a country with limited public health resources.

## Epidemiologic Profile of ECC (Prevalence)

Only two national epidemiologic studies on caries in primary teeth have been carried out in Peru. Both studies were designed by the National Center of Epidemiology, Prevention, and Disease Control of the Ministry of Health. The first study, conducted during 2001–2002, found that the prevalence of caries at age 6 years was 87.3% (*n* = 1,280), with an average dmft (decayed, missing, and filled teeth) of 6.7; the contribution of the decayed component was 6 of 6.7, revealing a high proportion of untreated caries (3). Although this study did not include younger children, results revealed that ECC has been a public health problem in Peru for more than 15 years (4).

The second national study found a caries prevalence of 76.2% in 3–5-year-old children (*n* = 2,195). The data were collected in all 25 Peruvian cities, across the three regions, between 2012 and 2014. The average dmft at this age range was five, with the decayed component contributing 4.5 to this score (4). Even though this study is the biggest ever conducted in the country and played an important role in better understanding of the extent and magnitude of ECC, the prevalence might be underestimated because WHO criteria were used and initial non-cavitated lesions were not considered. This methodologic problem was evident in three recent studies in Lima, which reported prevalence in two populations: those with and those without non-cavitated lesions (5–7). From those investigators' results, the prevalence of ECC is underestimated by about 11.9–24.8%, depending on the type of population. Severity measures, such as number of affected teeth, are also greatly underrated when cavities are the caries diagnostic threshold.

A selection of studies on ECC prevalence in Peru published in the last 10 years is shown in Table 1. More than 50% of the studies were conducted in children 3–5 years old with complete primary dentition. Although sample sizes were small, there were some important findings: Reported prevalence according to WHO criteria is above 70% in all studies, and average dmft is between 3.6 and 5 (6, 9, 11, 13, 14, 16, 17, 20). Data on younger children are sparse; only two studies have reported prevalence on children younger than 3 years (12, 15). Both studies included initial non-cavitated lesions in their criteria for assessment of caries, and they found a prevalence of 65.8 and 46.3% in 1–3 year-old and 6–36 month-old children, respectively. It is noteworthy also that, overall, higher prevalence was found in underserved groups, a finding that is supported by a recent systematic review of parental factors influencing ECC in developing nations, in which socioeconomic status was found significantly associated with ECC in 13 studies (21).

**Table 1:** Caries prevalence in children under 6 years old in Peru, 2010–2019.

**References**	**Type of Population** **(age)**	**Sample size**	**Caries criteria**	**Prevalence**	Average dmft^**^
Gonzales and Villena (5)	Preschoolers in a poor urban community in Lima (1–5 years)	230	ICDAS	87.2% 99.1%^*^	NR
Del Aguila and Isuiza (6)	Preschoolers in an underserved community in Loreto (3–5 years)	230	WHO	78.3%	NR
Olivera and Villena (8)	Preschoolers in an urban community in Lima (1–5 years)	230	ICDAS	59.1% 77%^*^	3.5 5.2^*^
Jiménez-Guillén and Cárdenas-Flores (9)	Preschoolers in a poor urban community in Lima (3–5 years)	92	WHO	72.8%	NR
Quiroga and Villena (7)	Preschoolers and babies during routine pediatric check-up in a poor urban community in Lima (1–5 years)	250	ICDAS	68.8% 93.6%^*^	5.4 10.6^*^
Huarachi and Barreda (10)	Schoolchildren in urban community in Arequipa (2–6 years)	192	NR	81.2%	4
Saravia and Macedo (11)	Preschoolers in an underserved community in Puno (3–5 years)	130	ICDAS	98.5%	NR
Clemente and Ortiz (12)	Routine pediatric check-up in mayor Hospital in Lima (6 months−3 years)	130	ICDAS II	46.3%	NR
Tobler et al. (13)	Preschoolers in an underserved community in Iquitos (3–5 years)	246	WHO	95.9%	5
MINSA (4)	Preschoolers from the three regions—National study (3–5 years)	2,195	WHO	76.2%	5
Muñoz-Luna (14)	Preschoolers in a poor urban community in Lima (3–5 years)	212	WHO	81.5%	NR
Cardenas-Flores and Perona-Miguel de Priego (15)	Schoolchildren in urban community in Ica (1–3 years)	231	WHO plus initial lesions	65.8%	NR
López-Ramos (16)	Preschoolers in a poor urban community in Huaura-Lima (3–5 years)	153	WHO	76.5%	3.6
Tamayo (17)	Preschoolers in a poor urban community in Lima (3–5 years)	90	WHO	85% (3 years) 90% (4 years) 92% (5 years)	3.6 (3 years) 4.3 (4 years) 5.9 (5 years)
Sanchez-Huamán and Sence-Campos (18)	Preschoolers in an underserved community in Lima (3 – 4 years)	623	WHO	70.6%	3.4
Villena et al. (19)	Routine pediatric check-up in poor urban communities in Lima (6 months−5 years)	332	WHO plus initial lesions	62.3%	3
Torres (20)	Schoolchildren in an underserved community in Lima (3–5 years)	246	NR (cavities)	83.7%	4.6

* *Initial non-cavitated lesions (white spot) included*.

** dmft- decayed, missing, filling per tooth.

*NR, Not reported*.

The studies on ECC in Peru, shown in Table 1, have limitations. Few of these reports have undergone a peer-review publication process; most are undergraduate theses published in online national repositories. Moreover, many of the reports did not describe the calibration process, and most did not report intra- and inter-Kappa coefficients. Another limitation is that the variation in levels of caries detection limits our ability to compare prevalence rates in cities in Peru and worldwide. Among the criteria used in national studies, we found these: WHO, WHO plus initial non-cavitated lesions, and International Caries Detection and Assessment System (ICDAS) (Table 1).

According to the article published by Kassebaum et al. (22), the age-standardized prevalence and incidence rates of untreated dental caries in deciduous teeth in 2010 for both sexes combined in the Andean region, which Peru is part of, was 8.3 and 14,470, respectively. Also, El Tantawi et al. reported that Peru is in the group of countries with highest prevalence of caries in children aged 36–71 months, between 75–100% (23).

Summarizing, dental caries in Peru is the most prevalent disease in school and preschool children and the main reason for external visits in primary healthcare centers. The prevalence and severity of this disease are dramatic, and they increase with age. Since Peru is among countries with the highest prevalence of caries in children in South America, urgent action is needed to reverse this situation.

## Risk Factors of ECC in Peru

According to the most recent definition of ECC, made during the ECC consensus meeting in Thailand, in 2018, dental caries is a dynamic and multifactorial disease, mediated by the biofilm and driven by sugar, that results in phasic demineralization and remineralization of the dental hard tissues. Dental caries also is determined by biological, behavioral, and psychological factors.

In this section, we will review the risk factors for ECC that have been studied in our country, considering that the number of studies is limited to specific groups, and extrapolation of the data to the whole population is difficult. We must remember that Peru is a diverse country with high disparities in oral health among the population.

### Sugars as a Risk Factor for ECC

Latin America has the highest sugar consumption per capita (41.8 kg/years in the 2015–2017 period) among developed and developing regions in the world (24). In Peru, the per capita consumption is about 38.2 kg/years (25). WHO guidelines recommend that intake of free sugars provide ≤ 10% of energy intake, with further reductions to <5% of energy intake throughout life to protect dental health (26). Peruvian children consume sugars from a very early age (27). According to a study by the National Institute of Health (INS), in 2015, children aged 6–36 months old have a high carbohydrate consumption, averaging 126.7 grams per day. Children consume diverse products that may produce a high risk for dental caries, such as sugar cane, which may be chewed habitually (28), and panatela, a combination of rice and sugars and other components. Parents sometimes add sugars or honey to liquids their babies consume to produce a better flavor, but the parents have little knowledge about the harm these practices do to the dental tissues. Villena and Bernal (29), found that 73% of children started eating sugars in liquid form before 6 months of age; 30% of children under 5 months of age consumed sugars an average of 2.5 times a day, and the rate increased to 4.6 times a day in children 1 year of age. In a study by Castillo et al. (30), most Peruvian children were found to like milk sweetened with some type of sugar, and, whereas only 44% of children liked plain milk, 96% liked sugared milk.

Many studies about the relationship of sugar consumption and ECC in children under 6 years old have been published in Peru. Unfortunately, many of these studies have methodologic problems and uncertainty about representation of the sample. Some studies have found a correlation between ECC and sugar consumption in various forms (27, 27, 31, 32). A study by Flores and Montenegro (33), however, found no relationship between the daily consumption of sugars and ECC, but all the patients were at high risk, which may have biased the results.

### Oral Hygiene

Peltroche et al. (34) found that among 3- to 6-year-old children with high caries risk, the most common factor was inadequate oral hygiene and visits to the dentist. Diaz et al. (35) found a relationship between poor oral hygiene and ECC in children under 3 years old. According to Rios (36) and Vega (37), the main tools used for oral hygiene in children under 6 years old was a piece of gauze or a toothbrush.

### Oral Health Literacy

According to Cupé-Araujo and García-Rupaya (38), 29.2% of Peruvian mothers of infants have deficient or irregular knowledge or oral health. Chambi (39) found that the level of oral health knowledge was poor in young mothers of children under 5 years old; those mothers did not receive any information about oral health when they were pregnant. Benavente et al. (40) found that even with a higher knowledge of oral health in mothers of children under 5 years old (77.6% of mothers had regular and good knowledge of oral health), ECC levels were high. Clemente and Ortiz (12) found that 55.4% of mothers of children under 3 years old did not receive any information about oral health.

### Nutrition and Dental Caries

Several studies on nutrition and dental caries have been conducted in Peru (41–43). The authors have found that one mild-to- moderate malnutrition episode during the first year of life is associated with increased caries in both the deciduous and permanent teeth. In another study, Delgado-Angulo et al. (44) found that stunting was a significant risk indicator for caries in permanent teeth over a 3.5-years period, independent of other well-known risk factors for caries development. Another study found no correlation between nutrition status and dental caries (45), although others (46) have reported a possible connection between malnutrition, enamel defects, and dental caries.

### Socioeconomic Factors

Several studies worldwide have found a relationship between dental caries and socioeconomic and education status. The only study that addressed this issue in Peru was that of Delgado-Angulo et al. (47), who found that poverty and social exclusion were associated with dental caries in primary and permanent dentition, and that children living in poor households had 2.25 times more risk for dental caries than did children living in non-poor households; although this study is the only one published regarding socioeconomic factors and ECC in Peru, the relation between poverty and ECC seems evident.

Despite the small sample size in many of the studies described above, which limits the generalizability of the results in the overall Peruvian population, many risk factors that influence the presence of ECC in Peruvian children have been established.

## Impact of ECC on Immediate and Long-term Growth, Development, Social, and Mental Health, and Well-being

During the ECC Summit in Thailand (2018), dental caries was described as common, usually untreated, and having a deep impact on children's lives. According to Martins et al. (48), dental caries is the main oral condition with the greatest impact on children's quality of life. Several studies have reported the impact of untreated dental caries on the quality of life of preschool children (49, 50). Lopez-Ramos and Garcia-Rupaya (16), using the Early Childhood Oral Health Impact Scale, validated in Spanish, found that ECC had a negative impact on oral health-related children's quality of life. However, there is little information published on the impact of ECC on the quality of life of Peruvian children.

A study by Diaz-Pizan (51) evaluated the quality of life related to oral health before and after dental treatment in children from Lima younger than 6 years of age. The average total score before treatment of children with ECC was 17.89, which decreased to 2.57 after treatment.

## Public Oral Health Interventions and Documents

Untreated dental caries is a huge burden for Peru's health economy (52). However, public health policies haven't given priority to oral health, probably because of the greater demands of life-threatening, prevalent diseases. Most community programmes have had no impact on reducing ECC prevalence in Peru, especially in the younger and underprivileged populations.

Fortunately, during the last few years, the Peruvian Ministry of Health has issued official documents on the importance for children's oral health (described below) (53). It remains to be seen if those documents will standardize oral health messages among health professionals and increase the use of protective factors during the first year of life.

### Community Programs

▘ In 1985, the Peruvian Ministry of Health approved a resolution to add fluoride to all commercially available salt (200 ppm of sodium fluoride per kg) (54). To date, there is no epidemiological data available on the effectiveness and/or employment of this resolution.
▘ In the year 2001, the Ministry of Health issued a guideline to regulate the use of glass ionomer cements for atraumatic restorative treatment. This guideline tried to offer a population approach to caries management with hand instruments (55).
▘ Other health strategies have been implemented in the past. They include the program “*Salud Escolar*” (Healthy Schools), started in 2013 and administered by the Health Promotion Department, which offers integral health evaluation and counseling to preschool and primary students in public schools. Oral examinations were performed on children, and basic oral health education was given to parents and teachers. The program intended to promote healthy habits in public schools (including toothbrushing) and provide a healthy environment (including healthy snacks in school kiosks) (56). Although the use of a common risk-factor approach is valid, no surveillance was performed. Thus, the positive outcomes of these programs have not been quantified and published.
▘ The main barriers to implementing preventive programs in Peru are the meager financial resources designated to children's health in general, but especially to oral health.

### New Documents

▘ In 2017, the Peruvian Ministry of Health issued the first Clinical Practice Guideline for the prevention, diagnosis, and management of caries in children below 11 years of age (57). (Eleven years is the age of the end of primary school education in Peru.) The recommendations of the Ministry are based on the best available evidence to assist practitioners and patients in making oral health decisions. The preventive recommendations (classified as grade A) include the regular use of standard fluoridated toothpaste from the time of eruption of the first tooth, the use of fissure sealants, and regular applications of fluoride varnish.
▘ In the same year, the Ministry of Health published the update of the Guidelines for the Healthy Growth and Development of Children below 5 years of age (58). The norm by which all nurse and health professionals abide states that oral health is a “priority issue” and that all children must be referred to a dentist or pediatric dentist before age one. The oral health appendix includes tables and pictures in concert with the national Clinical Practice Guidelines on dental caries; it comprises topics such as toothbrushing technique (lifting the lip); diagnosis of early caries lesions (white spots); control of sugar intake; correct use of fluoridated toothpaste (concentration and amount depending on age); and oral health recommendations based on the age of pre-schoolers. Educating and including other health professionals in oral health promotion may yield improved results in the oral health-related quality of life of Peruvian children.
▘ Other recent and important regulations developed by an alliance between the Peruvian Ministry of Health and the Ministry of Education will probably have a positive impact on oral health. The “Law on Healthy Diet” (59) and the “Implementation Manual on Food Advertising” (60) promote a healthier diet for all children and regulate commercial food labeling (black and white octagons). All food and beverages that contain high amounts of sugar, sodium or saturated fats, must indicate that in the corresponding octagons on the front of its package. The law was planned in two stages so that industries have time to adapt. The first stage is mandatory from June 2019. It states that all food products containing sugar ≥22.5 g/100 g and beverages containing sugar ≥6 g/100 ml should have a “High in Sugar” Octagon. The second stage, due September 2021, will require all food products containing sugar ≥10 g/100 g and beverages containing sugar ≥5 g/100 mL to have the “High in Sugar” Octagon. Moreover, an ordinance for “Healthy Kiosks” was passed, so that all public schools offer healthy food options for all students inside and outside their premises (61).

## Conclusions

Early childhood caries has recently been designated a global health problem (48, 62, 63). Despite limitations and variability across studies that have been conducted in Peru, we believe that Peru is amongst the countries with the highest prevalence of untreated ECC in the region, with negative consequences on the quality of life of preschoolers. High sugar consumption and oral health disparities (poverty, social exclusion, and low oral health literacy) may contribute to the high prevalence of ECC. From a research point of view, greater effort should be made to standardize methods in studies on ECC prevalence and related risk factors. It is also crucial to include children younger than 3 years old in national epidemiological studies, as was concluded in the recent WHO Global Consultation on ECC (63). Historically, public health policies have not given oral health high priority, but in the past 3 years, new regulations and evidence-based documents project a more favorable view on infant oral health in Peru.

## Author Contributions

JC has worked on the abstract, risk factors, quality of life, and conclusions sections. CP has worked on the abstract, prevention, quality of life, and conclusions sections. AC-M has worked on the epidemiology and conclusions section.

### Conflict of Interest

The authors declare that the research was conducted in the absence of any commercial or financial relationships that could be construed as a potential conflict of interest.
